# Nestin-expressing progenitor cells: function, identity and therapeutic implications

**DOI:** 10.1007/s00018-018-2794-z

**Published:** 2018-03-14

**Authors:** Aurora Bernal, Lorena Arranz

**Affiliations:** 1 Stem Cell Aging and Cancer Research Group, Department of Medical Biology, Faculty of Health Sciences, UiT, The Arctic University of Norway, MH Building Level 6, 9019 Tromsø, Norway; 2 Department of Hematology, University Hospital of North Norway, Tromsø, Norway; 3 Young Associate Investigator, Norwegian Center for Molecular Medicine (NCMM), Oslo, Norway

**Keywords:** Nestin, Central and peripheral nervous systems, Hematopoietic stem cell niche, Neural stem and progenitor cells, Mesenchymal stromal cells, Mouse models, Neurodegenerative diseases, Hematological malignancies

## Abstract

The neuroepithelial stem cell protein, or Nestin, is a cytoskeletal intermediate filament initially characterized in neural stem cells. However, current extensive evidence obtained in in vivo models and humans shows presence of Nestin^+^ cells with progenitor and/or regulatory functions in a number of additional tissues, remarkably bone marrow. This review presents the current knowledge on the role of Nestin in essential stem cell functions, including self-renewal/proliferation, differentiation and migration, in the context of the cytoskeleton. We further discuss the available in vivo models for the study of Nestin^+^ cells and their progeny, their function and elusive nature in nervous system and bone marrow, and their potential mechanistic role and promising therapeutic value in preclinical models of disease. Future improved in vivo models and detection methods will allow to determine the true essence of Nestin^+^ cells and confirm their potential application as therapeutic target in a range of diseases.

## Introduction

The neuroepithelial stem cell protein, more commonly known as Nestin, is a cytoskeletal protein classified as intermediate filament, initially described in neural stem cells (NSCs) of developing and adult brains [1, 2], and now known to be expressed in a variety of tissues and stem or progenitor cells, like pancreatic islets [3], skeletal muscle satellite cells [4], developing myotomes [5], testis [6], hair follicle [7], heart [8], and the non-hematopoietic fraction of the bone marrow [9–11]. Additionally, Nestin seems to play a role in pathogenesis and it is expressed in several types of malignancies, such as osteosarcoma, neuroblastoma, glioma, melanoma, pancreatic and prostate cancers as well as in tumor vasculature [12]. Further, Nestin is considered a biomarker of invasive phenotype, and is associated to infiltration in glioblastoma [13], angiogenesis in numerous malignancies [14] and spreading of non-epithelial and epithelial tumors [15]. This review focuses on the role of Nestin in the context of the cytoskeleton, the available in vivo models for the study of Nestin^+^ cells and their progeny, their function and nature in two major systems, neural and bone marrow, and their role and promising therapeutic value in preclinical models of disease of these two systems.

## Nestin: the stem cell intermediate filament

**Fig. 1:**
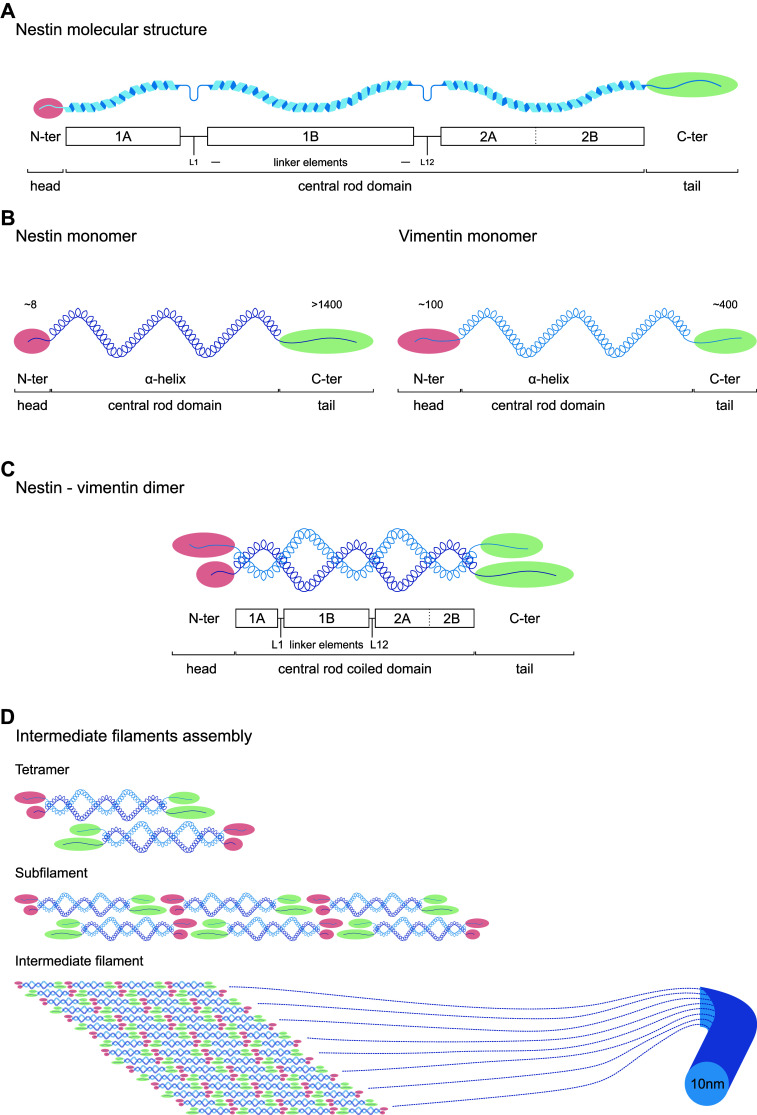
Nestin structure and assembly into intermediate filament. **a** Molecular structure of Nestin. Nestin shares a common structure with the other intermediate filaments that consists of a central α-helical rod domain (blue) of conserved size, flanked by a globular N-terminus (‘head’, red) and C-terminus (‘tail’, green) domains. The central α-helical rod domain consists of three segments separated by two linkers, i.e., coil 1A, linker 1, coil 1B, linker 12 and coil 2, with 2A and 2B and stutter between them. **b** Particularities of Nestin monomer. Nestin shows a short N-terminus (≈ 8 amino acids) and a long C-terminus (> 1400 amino acids) end, compared to other intermediate filaments like vimentin (≈ 100 and 400 amino acids, respectively). **c** Nestin forms heterodimers. N-terminus is required for intermediate filament assembly, and free C-terminus may interact with other cytoskeleton components. The short N-terminus disables Nestin to self-assemble into higher order structures, so Nestin needs other intermediate filaments to assemble, like vimentin. The central rod domain contains hydrophobic repeats (heptad repeats) that mediate dimerization allowing two α-helices to wrap around each other and become a ‘coiled coil’. Therefore, intermediate filament dimer rod central domain is known as central rod coiled domain. **d** Hypothetical intermediate filament assembly. Two dimers coil in antiparallel way in head-to-tail association to make a tetramer. Assembly takes place through rapid lateral aggregation of eight tetramers to form subfilaments, which assemble axially to form the intermediate filament (diameter ~ 10 nm). Numbers in N-termini and C-termini ends indicate number of amino acid residues. *N-ter* N-terminus, *C-ter* C-terminus

Nestin is classified as type VI intermediate filament, and constitutes a major component of the cytoskeleton. In contrast to general cytoskeletal elements, intermediate filaments are expressed in a cell-type specific manner in a way that major differentiation steps are marked by transition from one intermediate filament type to another. The complexity of intermediate filaments is remarkable, consisting of more than 50 distinct proteins able to form morphologically similar filaments in different cell subsets [16]. Polymerized intermediate filaments assemble through a highly conserved domain, whereas end domains of variable size and chemistry provide them with a wide variety of unique binding and regulating characteristics (Fig. 1) [16].

To date, six different types of intermediate filaments have been described (Table 1) according to their gene structure and protein sequence homology throughout their rod, head and tail domains. They include types I/II keratins, selective of epithelia; five type III proteins widely expressed, including vimentin expressed by mesenchymal cells; five type IV chains including neurofilaments and usually expressed only in neuronal tissues; several type V nuclear lamins expressed in many nucleated cells; and Nestin, a single type VI protein characteristic of a variety of stem cells, and NSCs in particular (Table 1). In terms of gene structure and protein sequence, the singularity of Nestin is unclear as it shows certain degree of similarity with type III and particularly type IV intermediate filaments [16]. Actually, it was proposed for reclassification as type IV intermediate filament based on its structure similar to neurofilaments [17]. However, due to differences in the α-helical region and its unique short N-terminus end and long C-terminus end (Fig. 1), it is still extensively considered as a separate entity [1, 16, 18]. In those classifications where Nestin is considered as a type IV intermediate filament, the type VI group includes two lens-specific proteins that bear little resemblance to other intermediate filaments and form beaded filaments: filensin and phakinin [19].

**Table 1:** Intermediate filament types, characteristic cell subsets and main function

IF type	Protein	No genes	Size (kDa)	Cell subsets	Function	References
I	Keratins (acidic)	> 25	40–60	K9–K20 and K23: soft epithelia; K31–K38: hard epithelia	Structural integrity, mechanical resistance, adhesion, signal transduction, inflammation, proliferation, apoptosis, motility	[116–119]
II	Keratins (basic)	> 24	50–70	K1–K8: soft epithelia; K81–K86, hard epithelia; K71–K74: inner root sheath		
III	Vimentin	1	53	Mesenchymal cells, developing neurons, astrocytes, endothelial cells, leukocytes	Structural maintenance, cell shape, motility, focal adhesion	[74]
	Glial fibrillary acidic protein	1	51	Neural stem cells, radial glia-like precursors, astrocytes, regenerative glia, mature glia in CNS and PNS	Structural maintenance, cell shape, motility	[2, 71–74, 120]
	Desmin	1	53	Satellite stem cells, striated muscle, replicating myoblasts, fibroblastic reticular cells of lymphoid organs	Contraction	[121, 122]
	Peripherin	1	58	Mature neurons in PNS	Neurite elongation	[123]
	Syncoilin	1	64	Skeletal and cardiac muscle, nervous system	Structural maintenance	[124]
IV	NF light	1	68	Maturing neurons in CNS, mature neurons in PNS and CNS	Functional maintenance, intracellular transport, morphogenesis of neurons	[125]
	NF medium	1	160			
	NF heavy	1	240			
	α-Internexin	1	66	Postnatal, maturing and mature neurons		[126]
	Synemin	1	230	Mature neurons in PNS, glia, skeletal and cardiac muscle	Structural maintenance	[74]
V	Lamin A/C	1	60–75	Nucleus, cell specific	Nuclear shape, chromatin scaffold, gene expression, differentiation, migration	[42, 127, 128]
	Lamin B1	1				
	Lamin B2	1				
VI	Nestin	1	240	Neural stem and progenitor cells, neurogenic cells, radial glia-like precursors, regenerative glia, mesenchymal stromal cells, subsets of endothelial cells, Schwann cell precursors in PNS	Self-renewal, proliferation, survival, differentiation, migration	[2, 9, 10, 20, 36, 47, 48, 50]

*K* keratin, *CNS* central nervous system, *PNS* peripheral nervous system, *NF* neurofilament

According to the *Human Protein Atlas*, Nestin is expressed in a variety of tissues including brain, bone marrow, immune system, muscle, lung, gastrointestinal tract, kidney and urinary bladder, adipose and soft tissues, female tissues and skin. Interestingly, mouse models have shown that enrichment of Nestin is evident and selective in several subsets of stem cells; yet it is unclear how Nestin influences stemness. Nestin^+^ cells from the adult brain are able to form neurospheres ex vivo and generate differentiated cells of neuronal and astrocytic lineages, which downregulate Nestin expression during differentiation [2]. Nestin^+^ cells from the adult bone marrow contain all the bone marrow colony forming unit fibroblastic activity and may be expanded as mesenspheres that self-renew in serial transplantations. Ex vivo, these cells can differentiate into osteoblastic, adipocytic and chondrocytic lineages, progeny that does not express Nestin [9]. Further, Nestin seems to be a marker of proliferation, given that most Nestin^+^ cells are also positive for the proliferation marker Ki-67 and are labeled by 5-bromo-2-deoxyuridine (BrdU) incorporation reflecting a proliferative state [2, 20]. This stands in contrast with Nestin^+^ periarteriolar cells from adult bone marrow that were found to be largely quiescent in vivo [21], divergence indicative of the potential cell-type specific roles for Nestin. Thus, Nestin is related to essential stem cell functions, including self-renewal/proliferation, differentiation and migration.

### Self-renewal/proliferation

The typical proliferative property of stem cells that ensures tissue homeostasis is asymmetric cell division, as it allows maintenance of the stem pool and generation of functional differentiated cells in parallel [22]. Asymmetric cell division leads to asymmetric inheritance of cellular components during cell division, which determines distinct cell fate for each daughter cell [23]; one of them retains self-renewal capacity and differentiation potential, whereas the other starts the differentiation path. This requires a series of profound morphological changes, including cytoskeletal arrangements that induce and maintain cell polarity.

The spindle apparatus is the cytoskeletal structure that forms during cell division to separate sister chromatids between daughter cells, and it includes mainly microtubules and associated proteins, like kinesin and dynein. Spindle orientation contributes to establishment of asymmetry in stem cell division and fate. For instance, it contributes to differential signaling environments in daughter cells [24]. In the developing cerebral cortex, horizontal divisions are mainly asymmetric and produce basal daughter cells that behave like young migratory neurons and apical daughter cells that remain within the proliferative zone, in contrast to vertical divisions that are preferentially symmetric and produce precursor cells. This was related to selective Notch1 inheritance by the basal neuron in horizontal divisions [25]. Further, spindle orientation defines nuclei position. In the early mouse embryo, as the 8-cell embryo progresses through the cell cycle, the nuclei of most cells move from apical to basal positions, in a microtubule- and kinesin-dependent manner [26]. Asymmetric cell divisions occur when nuclei are located basally, whereas cells showing apical nuclei divide symmetrically [26].

Hence, microtubules are well-known players in nuclear positioning, but the role of actin has also been highlighted. In the *C. elegans* embryo, mutations and chemicals affecting actin function disrupt partitioning-defective (Par) proteins asymmetry [27–29], which establish and maintain anterior–posterior polarity in the embryo [30]. Later, asymmetric inheritance of Par family proteins was demonstrated to contribute to the asymmetric outcome of neural glia divisions during development in mice [31, 32]. Further, in certain cell types like astrocytes, the retrograde flow of actin attracts major intermediate filaments, including Nestin, vimentin and glial fibrillary acidic protein (Gfap), in front of the nucleus, and thereby transfers pushing force that conditions nuclear positioning [33]. Using a neuronal progenitor cell line, ST15A, Sahlgren and colleagues showed that Nestin reorganization during mitosis is coupled to an increase in Nestin phosphorylation that contributes to partial disassembly of Nestin filaments [34]. Nestin may play a role in the regulation of assembly and disassembly of other intermediate filaments, like vimentin, during mitosis [35], and was proposed as mediator of the interaction between intermediate filaments with microtubules and/or microfilaments [17].

However, in *Nestin* knockout mice, the dramatic reduction in NSC survival and self-renewal occurs with no defects in microfilaments, microtubules or vimentin-based intermediate filaments, or changes in proliferation [36]. Surprisingly, a second *Nestin*^−*/*−^ mouse model was generated that showed grossly normal development of the central nervous system [37], although this work did not examine functional changes in NSCs. Unknown technical issues related to genetic engineering may underlie these unexpected differences. In both NSCs and ST15A cells, Nestin may protect from apoptosis by an independent mechanism that consists of binding to cyclin-dependent kinase 5; thereby inhibiting its proapoptotic function [36, 38]. Others have reported that Nestin is essential for proliferation of embryonic cortical neural progenitor cells. Small interference RNAs against Nestin were used to downregulate Nestin during rat brain development, resulting in G1 cell cycle arrest and reduced numbers of new neurons in vivo. Further, Nestin downregulation in cultured cortical neural progenitor cells inhibits their colony-formation capacity and causes a marked suppression in the phosphoinositide 3-kinase (PI3K) pathway under stimulation with growth factors. These effects are independent of vimentin, and can be rescued by up-regulation of PI3K activity [39]. Using the opposite strategy, Liu and colleagues showed that Nestin overexpression in vivo, in mouse embryos expressing a human Nestin cDNA transgene under the control of a ROSA26 promoter, leads to increased cell proliferation and activation of the PI3K and protein kinase B in heart and brain. No changes in apoptosis were reported [40]. Several reasons may have contributed to these discrepancies, including cell-type specific roles for Nestin as well as limitations in the technical strategies. However, it is interesting to hypothesize that presence of Nestin may be required for survival of the stem cell, whereas different levels of Nestin may have an impact on its proliferative status. Future work will be required for a better understanding of the specific role of Nestin in asymmetric cell division and stem cell self-renewal.

### Differentiation

As previously mentioned, asymmetric cell division and cytoskeletal rearrangements also imply a distinct morphology for one of the daughter cells that is committed to differentiation, and later the migration of mature cells, processes particularly evident during neurogenesis [41]. Cell differentiation is connected to changes in cellular shape based on intermediate filament remodeling, with several of these proteins co-expressed in a specific cell subset for a certain time during differentiation [17]. For instance, in the hematopoietic system, lamins determine the stem cell commitment to different cell lineages (Table 1). Lamins of type B predominate only in hematopoietic progenitors, erythroid differentiation is accompanied by high lamin A and low lamin B1 expression, and megakaryocytes do not develop under lamin suppression [42]. Nestin may be a good candidate to regulate differentiation, as its expression is enriched in several subsets of stem cells and down-regulated in committed cells [2, 9]. Surprisingly, to date studies are scarce and further, Nestin abolishment has no impact on neuronal differentiation during development in vivo [36]. Potentially, the massive cell death that occurs in the neural tube of *Nestin*^−/−^ embryos may mask other subtle yet relevant functions of Nestin. These would rather be evaluated using fine-tuned controlled levels of Nestin in time, like inducible mouse models. Yet during myogenesis, Nestin downregulation enhances differentiation, with no impact on proliferation of undifferentiated dividing myoblasts. In this system, Nestin prevents myoblast differentiation by inhibiting activation of cyclin-dependent kinase 5 through p25, crucial step in the process [43].

### Migration

Intermediate filaments are also key players in polarity maintenance in migrating cells [44, 45]. Migration of hematopoietic cell subsets through micropores is limited by the ratio of lamin A:B that also provides nuclear stiffness [42]. Vimentin has been extensively studied, and in wounded monolayers of retinal pigment epithelial cells, it interacts with microtubules to maintain cell polarization during healing [46]. Vimentin also regulates focal adhesion dynamics (Table 1). During development, Nestin deficiency does not influence neuronal movement or attachment of NSCs in vivo [36], but this function would be more adequately analyzed using fine-tuned inducible models.

Upon malignancy, growing body of evidence relates Nestin to improved ability of cancer cells to migrate and metastasize [15]. In prostate cancer cell lines, Nestin knockdown inhibits in vitro migration and invasion, with no effect on cell growth, and leads to a fivefold reduction of metastases compared to controls in spite of uncompromised tumorigenicity at the inoculation site [47]. However, in the same system, others have found that Nestin downregulation leads to a significant increase in phosphorylated focal adhesion kinase and integrin-dependent matrix degradation, and thereby to subsequent cell invasion [48]. Thus, Nestin may have different effects on cell function depending on cell subset, and transformation stage in case of malignancy.

## In vivo models for the study of Nestin^+^ cells and their progeny

**Fig. 2:**
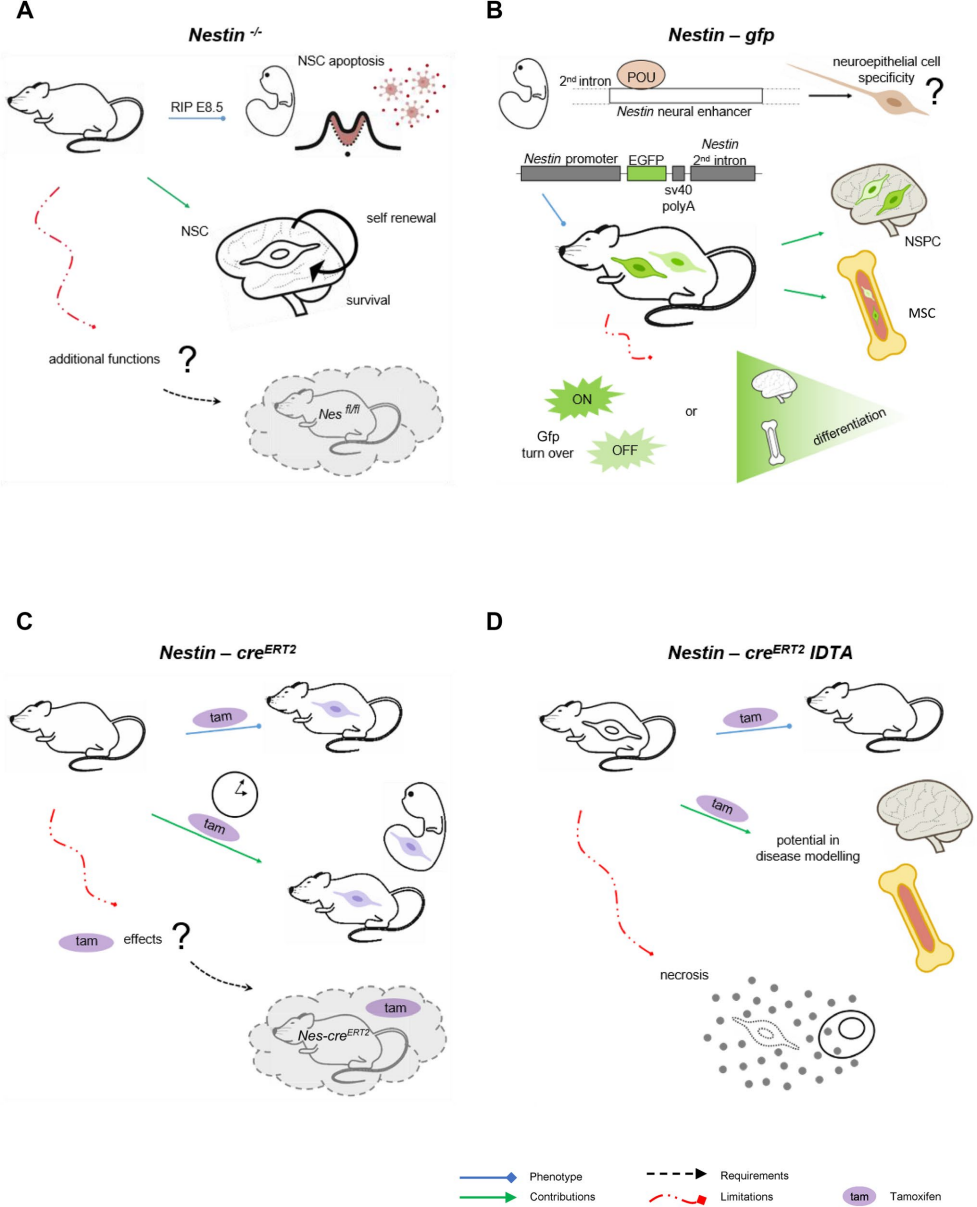
*In vivo* models for the study of Nestin^+^ cells and their progeny. **a** *Nestin*^−*/*−^ mouse model. *Nestin*^−*/*−^ mice are embryonically lethal (E8.5) due to neural stem cell apoptosis, which uncovered the role of Nestin in neural stem cell self-renewal and survival [36]. This model does not allow fine-tuned control of Nestin expression in space and time, so an improved strategy would be use of conditional and/or inducible knockout models, like *Nestin*^*fl/fl*^ intercrossed with cre lines of interest. **b** *Nestin*-*gfp* reporter mouse model. Pit-Oct-Unc transcription factors bind to the Nestin neural enhancer at the second intron to establish neuroepithelial cells specificity. *Nestin*-*gfp* mouse model was generated by selection only of these regulatory elements of the second intron and upstream area [2] in an attempt to direct *gfp* expression to neural stem cells. Later, presence of *Nestin*-*gfp*^+^ cells has been described in other tissues, particularly bone marrow. *Nestin*-*gfp*^bright^ and *Nestin*-*gfp*^dim^ cells have been reported with no robust evidence of differential and corresponding endogenous *Nestin* expression. In addition, *gfp* turnover may contribute to fluorescence intensity levels, which represents another limitation that may be overcome by fluorescent protein fusion to Nestin. **c** *Nestin*-*cre*^*ERT2*^ lineage tracing in vivo. This mouse model is based on cre technology, coupled to the modified estrogen ligand-binding domain (ERT2). The system activates under administration of tamoxifen, allowing tracing of Nestin lineage in a temporal manner, both in embryonic and adult stages. However, tamoxifen has physiological effects that must be distinguished using controls lacking *cre*^*ERT2*^ and treated with tamoxifen. **d** *Nestin*-*cre*^*ERT2*^ *iDTA* mouse model for Nestin lineage depletion. This mouse model is used to reduce numbers of Nestin^+^ cells and their lineage upon tamoxifen administration. It is useful to study their contribution to disease development. However, depletion of cells occurs through diphtheria toxin expression and subsequent necrosis, which implies release of cellular content and potential sterile inflammation. *Nes* Nestin, *Gfp* green fluorescent protein, *POU* Pit-Oct-Unc, *Tam* tamoxifen, *NSC* neural stem cells, *NSPC* neural stem and progenitor cells, *MSC* mesenchymal stromal cells, *iDTA* inducible diphtheria toxin A subunit

The generation of transgenic models has allowed to visualize and isolate Nestin^+^ cells through reporter genes, or to follow the fate of Nestin^+^ in vivo and genetically control their numbers and expression of additional genes, using the *cre* recombinase technology under the Nestin promoter (Fig. 2). The interest focused initially on NSCs in developing and adult brains [2, 20], but extended in recent years to other subsets of stem cells like those of the mesenchymal lineage [9]. Remarkably, in vivo models target selective, yet to a certain extent overlapping, Nestin^+^ cell subsets. Further, in spite of their high value, data derived from these tools should be considered in the context of their pitfalls as well. Hence, combination of models is preferred for complete answers to the questions under study.

### *Nestin*^−/−^

The first and most cited *Nestin*^−*/*−^ mouse model is embryonically lethal after E8.5 due to massive apoptosis of neural tube cells (Fig. 2a). This biological tool generated through targeting of the coding region of Nestin exon 1 by homologous recombination was essential to uncover the strong effect of Nestin on NSC survival and self-renewal [36], but it does not allow the study of additional functions of Nestin or its relevance at later stages. Surprisingly, Park and colleagues found no obvious abnormalities in other organs where Nestin^+^ cells are present, further supporting that Nestin function is indeed complex and cell-specific. Later, a second *Nestin*^−*/*−^ mouse model was generated by gene targeting, where the regions deleted included most exon 1 and part of the 5´ upstream region [37]. This is a less acknowledged *Nestin*^−*/*−^ mouse model that is viable, and show grossly normal development of the central nervous system but impaired motor coordination. The latter was attributed to aberrant neuromuscular junction areas with increased numbers of acetylcholine receptor clusters, nerve length, and endplate bandwidth [37]. Surprisingly, at the anatomical level, the authors reported no apparent morphological abnormalities resulting from the absence of Nestin expression by histological studies of the central nervous system or magnetic resonance imaging. However, these examinations could not rule out the possibility of subtle functional changes arising from the absence of Nestin in NSCs. Unknown technical issues related to the genetic engineering may underlie these puzzling differences that deserve further investigation. These may include generation of unidentified genetic defects in the first *Nestin*^−*/*−^ mouse model and/or emergence of a truncated protein with partial function in the second one. Further, in spite of the potential value of a conditional and/or inducible knockout mouse model for Nestin expression, *Nestin*^*fl/fl*^ mice have not been generated to date.

### *Nestin-gfp*

*Nestin* gene expression is regulated by enhancer elements residing in the intronic regions, and although Nestin is expressed in a variety of tissues, these regulatory elements show some selectivity for cell subsets. During embryonic development, members of the POU (Pit-Oct-Unc) transcription factor family bind to the Nestin neural enhancer at the second intron and establish neuroepithelial cell specificity [49]. By selection only of these regulatory elements of the second intron and the upstream flanking area, Mignone and colleagues [2] generated an essential reporter mouse model where they narrowed the spectrum of cells in which *gfp* is expressed and directed the fluorescence more specifically to NSCs (Fig. 2b). Later, low frequencies of *Nestin*-*gfp*^+^ have been reported in a variety of tissues, like bone marrow, where they represent mainly primitive perivascular mesenchymal stromal cells (MSCs) [9]. Interestingly, in fetal bone marrow, the majority of neural crest cells traced with *Wnt1*-*cre2* are *Nestin*-*gfp*^+^ cells that preserve MSC activity, but do not produce chondrocytes. Conversely, they help establish the hematopoietic stem cell (HSC) niche that supports HSC function. Neonatal *Nestin*-*gfp*^+^ cells also contain Schwann cell precursors that may be distinguished from MSCs by their absence of platelet-derived growth factor receptor α (Pdgfrα) [50]. Nevertheless, mature Schwann cells in the adult bone marrow do not seem to express *Nestin*-*gfp* [51].

The most remarkable limitation in this reporter line is represented by *gfp* turnover, which implies that fluorescence may be detected after *Nestin* expression is terminated. In several tissues, like brain and bone marrow, several *Nestin*-*gfp*^+^ subsets have been identified according to *gfp* levels [2, 21]. Surprisingly, bone marrow *Nestin*-*gfp*^bright^ cells were low for *Nestin* expression in samples analyzed by high throughput sequencing [21]. In addition, although Gfap^+^ Schwann cells in the adult bone marrow do not express *Nestin*-*gfp* [51], both markers colocalize when Nestin is detected by antibody [52]. Limitations in *gfp* detection methods, Nestin antibodies and stromal cell obtaining protocols may contribute to these inconsistencies. Further, using distinct transgene constructs and/or control under different regulatory elements, other *Nestin*-*gfp*^+^ mouse models have been generated that differ in *gfp* patterns [53]. Thus, *gfp* expression may not totally reproduce endogenous *Nestin* expression.

### *Nestin*-*cre*/*Nestin*-*cre*^*ERT2*^

*Cre* mediated recombination is a broadly used genetic tool to conditionally delete or express selected genes, and to lineage trace progenitor populations during development when combined to a reporter gene with an upstream floxed-stop cassette [54]. Several *Nestin*-*cre* lines have been generated [55, 56], and used to direct recombination to neural stem and intermediate progenitor cells [57]. However, the rate of *Nestin*-*cre* driven recombination may reach sufficient levels in NSCs and progenitors only during late embryonic and early postnatal stages [58]. Further, in *Nestin*-*cre* models, recombination occurs in Nestin^+^ cells and is maintained in their progeny. This allows lineage tracing of Nestin^+^ cells but represents a drawback in studies restricted to Nestin^+^ cells only, given that their progeny of differentiated cells cannot be ruled out. This may underlie controversial findings like presence of recombination in fully differentiated neurons [59] or their poor colony forming unit fibroblastic activity in adult bone marrow [60].

In addition to central/peripheral nervous system and bone marrow, *Nestin*-*cre* is expressed in a variety of tissues including kidney, heart, lung, muscle, intestine, pancreas, spleen and skin. Besides, genomic insertion of *cre* may influence physiological function due to random integration that may disrupt endogenous gene expression and/or to illegitimate recombination catalysis, among others. Potentially, this may explain the marked metabolic phenotype reported in *Nestin*-*cre* mice with no targeted gene. Thus, *Nestin*-*cre* controls are necessary in experimental designs to prevent misinterpretation of results [61].

*Nestin*-*cre*^*ERT2*^ is an improved genetic approach to control Nestin lineage in a temporal manner [62], which is particularly relevant for studies on adult Nestin^+^ cells (Fig. 2c). This transgenic mouse model expresses a fusion protein encoding *cre* coupled to the modified estrogen receptor ligand-binding domain (*ERT2*). *Cre*^*ERT2*^ is silent but activates upon estrogen analog tamoxifen administration. Duration of tamoxifen treatment influences efficiency of recombination, which needs to be considered when interpreting data obtained with this model, as well as when making comparisons among studies. Administration of tamoxifen in neonatal *Nestin*-*cre*^*ERT2*^ mice promotes higher recombination efficiency than in adults [50]. In addition, tamoxifen has significant effects on cell function and physiology [63] that cannot be distinguished unless use of appropriate experimental controls lacking *cre*^*ERT2*^ and treated with tamoxifen. Further, potential interactions between tamoxifen and the selective genetic manipulation in Nestin cell lineage may occur and render data interpretation more difficult.

### *iDTR/iDTA*

*Cre* technology under the Nestin promoter allows depletion of Nestin^+^ cells in vivo, when intercrossed with the *cre* recombinase-inducible diphtheria toxin receptor line (*iDTR*) [64]. In adult *Nestin*-*cre*^*ERT2*^ *iDTR* mice, tamoxifen and diphtheria toxin treatment severely reduces the numbers of bone marrow Nestin^+^ cells [9]. To date, however, it remains unclear whether both subsets of adult bone marrow Nestin^+^ cells, *Nestin*-*gfp*^bright^ and *Nestin*-*gfp*^dim^ cells, are equally depleted by this strategy or to what extent. Further, ablation of Nestin^+^ cells may occur in additional organs, like brain, which may have an indirect impact on hematopoiesis and complicate interpretation of results. Mouse cells are significantly more resistant to diphtheria toxin than human cells, mainly due to three amino acid changes in DTR involved in binding of diphtheria B subunit [65]. However, some degree of toxicity from diphtheria toxin treatment cannot be discarded.

An improved approach is the *cre* recombinase-inducible diphtheria toxin mouse line (*iDTA*) that expresses the toxic diphtheria A subunit [66] (Fig. 2d). In adult *Nestin*-*cre*^*ERT2*^ *iDTA* mice, treatment with tamoxifen does not affect mature bone marrow Schwann cells, but reduces the numbers of Nestin^+^ MSCs [51]. The utility of this model is outstanding, but potential unspecific effects are not accurately defined. Diphtheria toxin causes necrosis by inactivation of elongation factor 2 through adenosine di-phosphate ribosylation, thereby preventing protein synthesis. Minor leakage in expression may be detrimental given the high toxicity of DTA, efficient to arrest translation as a single molecule inside the cell [67]. Further, necrosis implies release of cellular content. The *iDTA* system lacks the B subunit of the toxin responsible for binding to the receptor, so no detrimental effects are expected from DTA discharge. However, subsequent sterile inflammation at the site of cell death may occur and impact phenotypes under study.

## Identity of Nestin^+^ cells

**Fig. 3:**
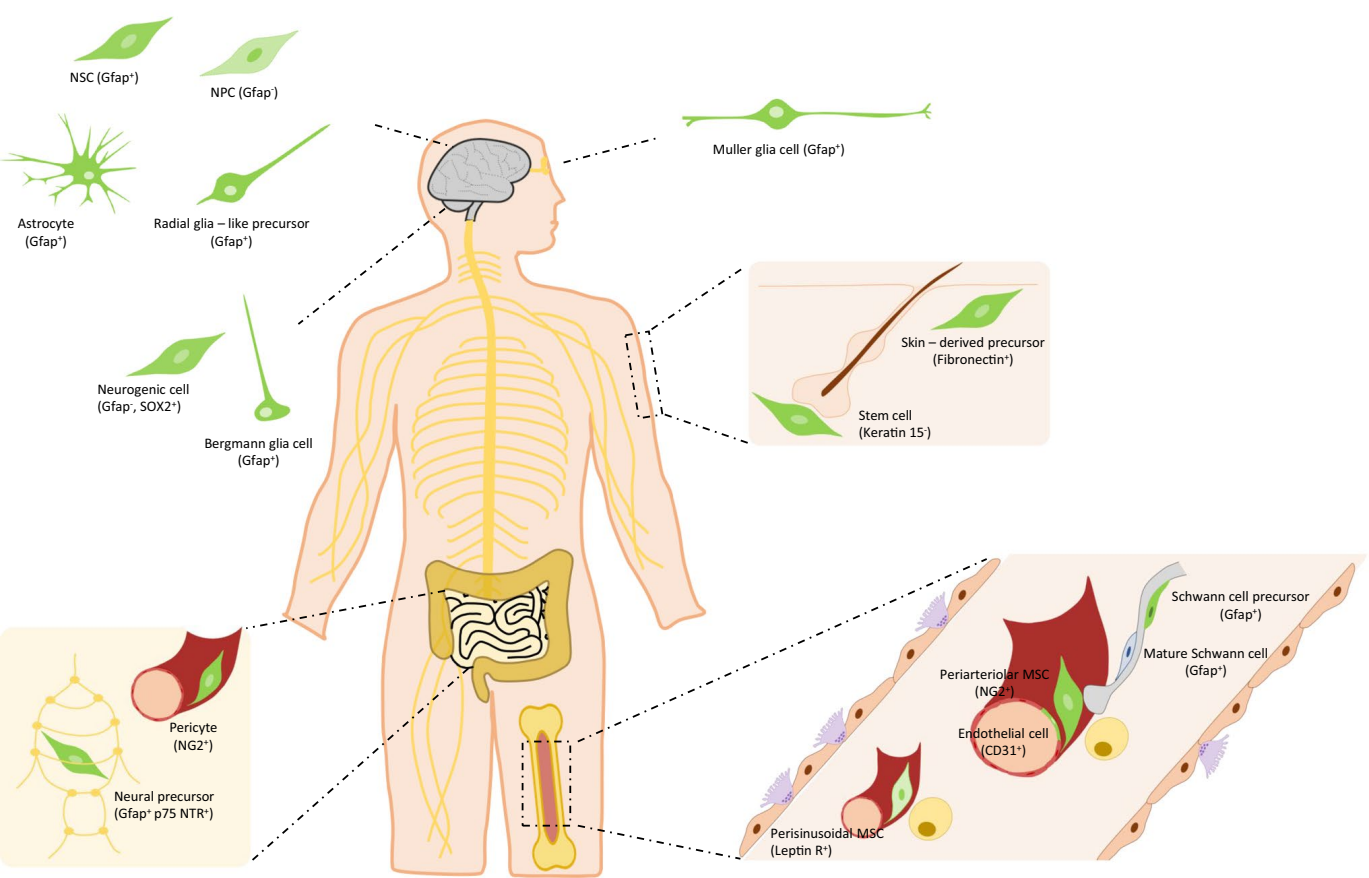
Identity of Nestin^+^ cells found in neural tissues and bone marrow. The main subsets of Nestin^+^ cells present in neural-related tissues (brain, cerebellum, retina, gut, skin) and bone marrow are shown. These subsets comprise neural stem cells, neural progenitor cells, astrocytes, radial glia-like precursors, subsets of glia cells with regenerative potential, several types of neural precursor cells outside brain, pericytes, mesenchymal stromal cells, endothelial cells, and Schwann cell precursors. They are distinct in their location, functional properties and co-expression of selective markers. Nestin^+^ cells are represented in green. In brain and bone marrow, *Nestin*-*gfp*^bright^ and *gfp*^dim^ cells have been described according to gfp intensity levels in *Nestin*-*gfp* mice. *NSC* neural stem cells, *NPC* neural progenitor cell, *MSC* mesenchymal stromal cell, *Gfap* glial fibrillary acidic protein, *SOX2* sex determining region Y-box 2, *p75 NTR* p75 neurotrophin receptor, *NG2* neural/glial antigen 2, *Leptin R* leptin receptor

The above transgenic models have helped identify different subsets of Nestin^+^ cells, depending on factors like location and time (developmental or adult stage), among others. In this section, we discuss the comprehensive literature on the main subsets of Nestin^+^ cells characterized to date in two major locations, nervous system and bone marrow (Fig. 3).

### Central and peripheral nervous systems

In the developing brain, the regulatory elements at the second intron direct *gfp* expression in *Nestin*-*gfp* mice to the neuroepithelium of the embryo where Nestin is expressed [2, 5, 49]. In the adult brain, *gfp* is expressed widely in the areas where Nestin expression is found, like the lateral wall of the lateral ventricle, olfactory subependyma and dentate gyrus, characterized by persistent production of new neurons, whereas it is absent both in differentiated cells and in areas containing fully differentiated cells. *Nestin*-*gfp*^+^ cells of the rostral migratory stream comprise two subsets according to gfp intensity; *gfp*^bright^ cells are positive for Gfap and *gfp*^dim^ cells co-express III-tubulin. These two subsets were suggested to be progenitor cells at different stages of differentiation, where *gfp*^bright^ cells would be stem cells and *gfp*^dim^ cells would represent more mature precursors [2]. Moreover, most of the proliferating cells are also *gfp*^+^. In the proximal part of the rostral migratory stream, the majority of BrdU^+^ nuclei is found among *gfp*^bright^ cells, whereas in distal areas, close to the olfactory bulb, most BrdU^+^ cells have low levels of gfp. This further supports that *gfp* levels distinguish subsets of neuronal progenitors in the adult brain [2]. However, *Nestin* expression was not confirmed in sorted *gfp*^bright^ and *gfp*^dim^ cells, and gfp levels may not exactly mirror expression of the endogenous *Nestin* gene.

As previously discussed, *Nestin*-*gfp*^+^ cells are enriched in neurosphere-forming cells, and actually most of the neurosphere-forming cells of the adult brain are contained within *gfp*^+^ cells. The neurospheres generated from individual *gfp*^+^ cells contain a large number of *gfp*^bright^ cells, with certain degree of heterogeneity in gfp levels. When the neurospheres are plated on laminin substrate, fluorescence declines dramatically and most cells express markers of differentiated cells like III-tubulin, Gfap, or 2′,3′-Cyclic-nucleotide 3′-phosphodiesterase [2].

Interestingly, most Nestin^+^ cells express Gfap, although co-expression is dependent on species and developmental stage. In primates, radial glia cells in the developing neocortex express Gfap from the initiation of neurogenesis, in contrast to rodents where Gfap is expressed after birth [68–70]. In addition, both Nestin and Gfap are expressed in the subclass of radial glia-like precursors in the adult mouse dentate gyrus [71]. Both markers are present in Muller glia cells that differentiate to neural progenitor/stem cells to regenerate lost photoreceptors and neurons in the retina under pathological conditions [72], and in cerebellum Bergmann glia cells which express the transcription factor sex determining region Y-box 2 (Sox-2) [73]. Besides Gfap and Nestin, astrocytes and astrocytoma cells express vimentin and possibly synemin [74] in a dense filament network [45]. The integrity of this network is required for correct nuclear positioning, microtubule organization, and cell polarity [33, 45]. Recently, another potential neurogenic cell subset has been identified in the adult cerebellar cortex that expresses Sox-2 and *Nestin*-*gfp*, and lacks markers of differentiation like Gfap and S100β [75].

In the periphery, a subset of Nestin^+^ cells give rise to adult myenteric neurons in gut, suggesting that they are neural precursors cells [20]. *Nestin*-*gfp*^+^ cells form a network distributed through most of the wall of the small intestine. They are particularly abundant in the submucosal zone and muscular layers, but not present in the epithelial lining. This network is mainly perivascular, but small numbers of cells are also found in the myenteric plexus [20]. The potential neural crest origin of *Nestin*-*gfp*^+^ cells was traced with *Wnt1*-*cre* together with a reporter, which labels *Nestin*-*gfp*^+^ cells in the myenteric plexus but not the perivascular compartment. Perivascular *Nestin*-*gfp*^+^ cells express the pericyte marker nerve/glial antigen 2 (NG2). Conversely, *Nestin*-*gfp*^+^ cells in the myenteric ganglia may express p75 Neurotrophin receptor (p75Ntr), CD49b, Gfap and S100β, but do not express the neuronal marker protein gene product 9.5. Although limited, the neurosphere-forming capacity of myenteric cells is restricted to *Nestin*-*gfp*^+^ cells (~ 3.5%) and particularly to those that coexpress p75Ntr (~ 10%). Neurospheres differentiate in vitro to neurons and glia, and cells traced with *Nestin*-*cre*^*ERT2*^ in vivo include both HuC/D^+^ neurons and S100β cells. Surprisingly, expression of the reporter in this mouse line correlates with endogenous Nestin expression in the plexus. Further, cells isolated by the reporter also form neurospheres that differentiate into neurons in vitro, and engraft and differentiate to HuC/D^+^ neurons in vivo upon transplantation into the wall of recipient mice. *Sox10*-*H2BVenus* mice and immunostaining with Nestin antibody was used to rule out the potential glial nature of *Nestin*-*gfp*^+^ [20]. However, as previously discussed, these two methods may detect only partially overlapping cell subsets.

In skin, a subset of adult stem cells, namely skin-derived precursors, was identified under culture adapted from generation of NSCs from brain [76]. This small subset of skin cells from adult mice form spheres, which dissociated and plated onto poly-d-lysine/laminin express Nestin in vitro. Skin-derived precursors can differentiate in vitro into neurons, glia, smooth muscle cells and adipocytes. Unlike NSCs, skin-derived precursors express fibronectin. Further, skin-derived precursors do not express p75Ntr nor polysialic acid-neural cell adhesion molecule, and they did not differentiate into tyrosine hydroxylase^+^ neurons. Similar cells were found in adult human skin [76]. More recently, skin-derived precursors showed differentiation in vitro into tyrosine hydroxylase and dopamine-β-hydroxylase neurons [77]. Single spheres generated from the skin of neonatal mice expressing yellow-fluorescent protein (Yfp) from the actin promoter transplanted into the chick neural crest migratory stream *in ovo* showed migration into peripheral neural crest targets and dermis. Nestin^+^ cells are found in mouse skin, and neural crest derived cells traced with *Wnt1*-*cre* in vivo are present in the dermal papilla of the hair follicle [77]. However, the correspondence between both cell subsets was not confirmed. Further, the potential overlap between skin-derived precursors and MSCs remains unclear. Later, *Nestin*-*gfp*^+^ cells were observed in the hair follicle bulge [7]. They behave as stem cells, giving rise to much of the hair follicle during hair growth like the outer root sheath and the interfollicular vascular network. *Nestin*-*gfp*^+^ cells are negative for keratin 15 and are capable of differentiation into neurons, glia, keratinocytes, smooth muscle cells and melanocytes in vitro. Further, *Nestin*-*gfp*^+^ cells may give rise to neurons after in vivo transplantation to the subcutis of nude mice [7]. In humans, Nestin^+^ cells that can be propagated and exhibit multipotent potential in vitro are also found in other locations, like eccrine and apocrine sweat glands of axillary skin [78], but it is unknown where they originate from.

### Bone marrow

Adult HSCs have the capacity to both self-renew and differentiate into all blood cell lineages, being responsible for blood production during the entire life [79]. The HSC niche is a dynamic entity that supports the function, fate and numbers of HSCs in the bone marrow where these cells reside [80]. MSCs are relevant components of the HSC niche, yet the absence of selective markers to track them in vivo has limited our understanding of their nature [81]. Further, limitations in genetic tools for the study of MSCs have contributed to discrepancies among studies [82].

In this scenario, *Nestin*-*gfp*^+^ cells in adult bone marrow were initially identified as MSCs that contain all the bone marrow colony forming unit fibroblastic activity and expand as mesenspheres that self-renew in serial transplantations. *Nestin*-*gfp*^+^ cells were described as perivascular cells closely associated to HSCs and sympathetic fibers, and enriched in expression of HSC maintenance genes. In this study, no *gfp*^+^ cells were found within the endothelial CD31^+^ fraction in the bone marrow by microscopy or flow cytometry. Ex vivo, *Nestin*-*gfp*^+^ cells differentiate into osteoblastic, adipocytic and chondrocytic lineages, cells who do not express Nestin. In vivo, using *Nestin*-*cre*^*ERT2*^ intercrossed with a reporter line, *gfp*^+^ osteoblasts, osteocytes and chondrocytes were traced after 8 months chase with tamoxifen. In vivo depletion of Nestin^+^ cells and their progeny using *Nestin*-*cre*^*ERT2*^ *iDTR* mice reduced HSCs numbers by four-fold, 1 month after administration of tamoxifen and diphtheria toxin [9].

Later, using whole-mount confocal immunofluorescence and computational modelling, two distinct *Nestin*-*gfp*^+^ cell subsets were identified according to fluorescence intensity by microscopy, *Nestin*-*gfp*^bright^ cells are rare and found exclusively along arterioles and *Nestin*-*gfp*^dim^ cells are reticular in shape and associated with sinusoids. *Nestin*-*gfp*^bright^ cells are enriched in colony forming unit fibroblastic activity and in the expression of HSC maintenance genes. Further, dormant HSCs localize close to *Nestin*-*gfp*^bright^ cells and this protects them against genotoxic insults [21]. However, HSC maintenance and colony forming unit fibroblastic activity were traditionally related to perisinusoidal cells [83, 84]. Moreover, leptin receptor (Lepr)^+^ perisinusoidal cells traced cells using a constitutive *cre* system showed that these cells are an important source of stem cell factor, critically support HSC maintenance and partially overlap with *Nestin*-*gfp*^+^ cells [85]. *Nestin*-*gfp*^dim^ cells were indeed confirmed to largely overlap (~ 80%) with *Lepr*-*cre* cells, whereas *Nestin*-*gfp*^bright^ cells represent a distinct cell subset that is positive for the pericyte marker NG2 and α-smooth muscle actin. Using *NG2*-*cre*^*ERTM*^ induced by tamoxifen together with a reporter mouse line intercrossed with *Nestin*-*gfp* mice, 30% of *Nestin*-*gfp*^bright^ cells were labeled with the reporter but no labelling was found within *Nestin*-*gfp*^dim^ cells. Depletion of NG2^+^ cells in *NG2*-*cre*^*ERTM*^ *iDTR* mice reduced *Nestin*-*gfp*^bright^ and the HSC pool [21]. However, conditional deletion of stem cell factor or Cxcl12 in *NG2*-*cre*^*ERTM*^ traced cells has no influence of the HSC pool [86], although a longer tamoxifen administration protocol to improve the efficiency of Cxcl12 deletion led to a significant reduction of the CD150^+^ CD48^−^ HSC pool [87]. In contrast, *NG2*-*cre* cells stain for Lepr and label both *Nestin*-*gfp* subsets, osteocytes, chondrocytes, adipocytes and a small fraction of endothelial cells (~ 10%) [87]. Whereas deletion of Cxcl12 in *Lepr*-*cre* targeted cells leads to mobilization of HSCs [88], the same strategy in *NG2*-*cre* cells leads to both mobilization and quiescence exit [87]. Moreover, *NG2*-*cre* but not *NG2*-*cre*^*ERTM*^ targeted cells seem to be a relevant source of stem cell factor [87]. This may be explained on the basis of their large overlap with Lepr^+^ perivascular cells and maybe endothelial cells, both of which demonstrated as the major sources of stem cell factor in the bone marrow [85]. Hence, the nature of bone marrow mesenchymal *Nestin*-*gfp*^+^ cells, and in particular *Nestin*-*gfp*^bright^ cells, remains controversial. These studies highlight the complexity of in vivo models and emphasize the importance of thorough consideration of experimental conditions for adequate data interpretation in the context of the bone marrow niche.

Using a milder enzymatic protocol that allows better preservation of antigen expression, *Nestin*-*gfp*^+^ CD31^+^ endothelial cells are detected in the fetal bone marrow, and their number increase with age [50]. This fact points out another major source of discrepancies among studies derived from different enzymatic digestion protocols, required for obtaining the stromal fraction of the bone marrow. Recently, the group of Lapidot described the existence of *Nestin*-*gfp*^+^ cells within the adult non-hematopoietic (CD45^−^) endothelial (CD31^+^) bone marrow fraction, and particularly restricted to Sca-1^+^ CD31^high^ arterial bone marrow endothelial cells. Interestingly, bright *Nestin*-*gfp*^+^ Sca-1^−^ MSCs are adjacent to *Nestin*-*gfp*^+^ Sca-1^+^ endothelial cells, and *Nestin*-*gfp*^+^ NG2^+^ MSCs are closely associated to *Nestin*-*gfp*^+^ NG2^−^ blood vessels [10]. However, the potential lineage relationship between *Nestin*-*gfp*^+^ MSCs and *Nestin*-*gfp*^+^ endothelial cells in the adult bone marrow remains unknown. In the embryonic perichondrium, *Nestin*-*gfp*^+^ comprise endothelial and non-endothelial cell subsets that interact with each other and increase their numbers during vascular invasion and endochondral ossification. Further, in the developing bone marrow, cells traced with *Nestin*-*cre*^*ERT2*^ in vivo are predominantly endothelial cells, but encompass osteoblasts, osteocytes, stromal cells and chondrocytes as well [11]. Of note, different layers of endothelial and perivascular *Nestin*-*gfp*^+^ cells may make arterioles appear brighter under the microscope, which raises the possibility that arteriolar *Nestin*-*gfp*^bright^ cells under the microscope may not completely overlap with the brightest cells isolated by FACS as single cells [50]. Moreover, fluorescence intensity thresholds may not be fully comparable between both methods.

In fetal bone marrow, most neural crest cells traced with *Wnt1*-*cre2* are *Nestin*-*gfp*^+^ cells that preserve MSC activity, but do not produce chondrocytes. Conversely, they support HSC function. Neonatal *Nestin*-*gfp*^+^ cells also contain Gfap^+^ Schwann cell precursors that differ from *Nestin*-*gfp*^+^ MSCs in lack of Pdgfrα [50]. By immunohistochemical and ArrayScan analyses of the adult bone marrow, non-myelinating Schwann cells positive for Gfap were found positive for Nestin and negative for Pdgfrα, all through antibodies. Further, Pdgfrα^+^ MSCs and Nestin^+^ cells largely overlap but show different distributions [52]. Gfap^+^ Schwann cells are in close association to arteries, but unfortunately colocalization of gfp and Gfap in *Nestin*-*gfp* mice was not confirmed [10]. Further, *Nestin*-*gfp*^bright^ cells are in direct contact with Gfap^+^ Schwann cells, but both markers were not reported to colocalize [21]. Hence, the relation between Schwann cells and *Nestin*-*gfp*^+^ cells in adult bone marrow is yet to be elucidated. Interestingly, using an in vivo model of hematopoietic malignancies, namely myeloproliferative neoplasms, we showed that both cell types are reduced in the disease bone marrow which contributes to pathogenesis. *Nestin*-*gfp*^+^ cells from disease mice are reduced in the expression of MSC and HSC niche-related genes, but enriched in Schwann cell genes and neural-related functional categories. Principal component analyses of genomic data confirmed that *Nestin*-*gfp*^+^ cells from disease mice cluster away from primitive MSCs and close to Schwann cells. However, Gfap^+^ Schwann cells in the bone marrow do not seem to express gfp in *Nestin*-*gfp* mice, although both cells subsets are closely associated spatially. Additionally, selective depletion of Nestin^+^ in vivo using *Nestin*-*cre*^*ERT2*^ *iDTA* mice did not affect Schwann cells but reduced numbers of CD90^+^ mesenchymal cells [51]. Previously discussed factors related to the in vivo tools, like gfp patterns and efficiency of recombination, may have contributed to these intriguing results that should be subject of future research.

## Role of Nestin^+^ cells in pathogenesis and value of its targeting in therapeutic strategies

Recent body of evidence on Nestin and its role in tissue remodeling and cell regulation of other cell subsets, and particularly stem cells, suggests that Nestin may have great potential as target to develop new therapeutic strategies in the clinic and/or tools with diagnostic/prognostic value in patients.

### Nervous system

Underlying the greatest challenge in finding cures for neurodegenerative diseases there are several causes, including not fully understood driving forces and disease onsets far long prior to the stage of detectable symptoms. As previously discussed, compiled evidence supports Nestin as a good marker of NSCs and progenitors, so efforts are currently being directed to study its contribution in mechanisms of disease and its targeting for development of novel therapeutic strategies and/or diagnostic/prognostic methods in diseases of the nervous system, particularly those with degenerative component.

Alzheimer’s disease is characterized by progressive deposition of β-amyloid peptides in a variety of brain regions, and concomitant neuronal and synaptic loss as well as defective neurogenesis [89]. Presence of the ε 4 allele in apolipoprotein E (ApoE) represents the most important genetic risk factor for disease development [90]. Surprisingly, to date few molecules are described to play regulatory roles in adult neurogenesis with relevance to Alzheimer’s disease [91]. Using an ApoE-deficient mouse model intercrossed to *Nestin*-*gfp* mice, lack of ApoE increased proliferation of early NSCs and progenitors within the dentate gyrus, resulting in eventual depletion of the overall early progenitor pool that coexpresses Nestin and Gfap [92]. Recently, mouse NSCs were isolated on the first day of birth, cultured in vitro with retinoids acid to induce differentiation, and transplanted bilaterally at the hippocampus of amyloid precursor protein and presenilin-1 transgenic mice [93]. Although scarce information is provided on the nature of the stem cells used for therapy, fractions of cells were reported to express differentiation markers in vivo, including Gfap or microtubule-associated protein 2. This strategy restores the reduced numbers of Nestin^+^ cells and promotes proliferation in dentate gyrus and subgranular zone, and increases numbers of synapses in the transplanted regions. The authors propose that resulting neurogenesis may be monitored by proton magnetic resonance spectroscopy in vivo, which in turn would be proportional to Nestin^+^ cell improvements [93]. Future work should elucidate the value of Nestin^+^ cells as potential therapeutic target and/or prognostic parameter with improved methods in patients of Alzheimer’s disease.

Hence, efforts are dedicated to improve the molecular imaging of Nestin in real time in vivo. Recently, a transgenic mouse model was generated bearing both luciferase and gfp under control of the Nestin promoter. This system allows to trace *Nestin* transcription in living animals using both bioluminescence and fluorescence, and a high resolution camera [94]. Interestingly, upon middle cerebral artery occlusion and lipopolysaccharide-induced innate immune system activation as models of stroke and neuroinflammation, respectively, Nestin expression patterns seem to shift from progenitors towards activated microglia/macrophages and astrocytes. Direct functional consequences of this shift are yet to be characterized but these data suggest that Nestin may serve as biomarker of inflammatory responses in brain [94]. Indeed, development of improved non-invasive imaging tools for Nestin follow-up in patients will be highly relevant in the clinic.

Amyotrophic lateral sclerosis results from progressive motor neuron degeneration leading to impaired motility. The most frequent genetic cause is a hexanucleotide repeat expansion (GGGGCC) (over hundreds) in a noncoding region of C9ORF72, protein found in neurons and presynaptic terminals suspected to participate in protein trafficking. This mutation is also common in familial frontotemporal degeneration, a related but different neurodegenerative disease [95]. Both gain- and loss-of-function mechanisms have been proposed. Recently, C9orf72 loss-of-function in Nestin^+^ cells and their lineage was tested by intercrossing of *C9orf72*^*fl/fl*^ and *Nestin*-*cre* mouse lines [96]. Although this is a broad strategy that involves absence of C9orf72 in neural progenitors, neurons and glial cells from developmental stage, it is interesting to see that resulting mice lack hallmarks of amyotrophic lateral sclerosis, like reduction of motor neurons, astro- and microgliosis, and ubiquitination enhancement [96]. Hence, these data may have implications for development of therapies, as they may indicate that strategies targeting the repeat expansion that also reduce C9ORF72 expression are unlikely to have negative secondary effects due to the latter.

Parkinson’s disease is the result of massive degeneration of dopamine neurons in the midbrain, and particularly the substantia nigra, which causes motor symptoms like resting tremor, and other symptoms including neuropsychiatric [97]. Further, dopamine modulates neurogenesis that is then impaired in Parkinson’s disease patients [98]. *Nestin*-*cre*^*ERT2*^ intercrossed with reporter mouse lines have been used to study the neurogenic capacity of Nestin^+^ cells in midbrain under steady state conditions. Interestingly, *Nestin*-*cre*^*ERT2*^ -traced cells are in low frequencies and show mature neuronal phenotypes even after short-term tamoxifen treatments, but their function and gene expression differ from neighboring cells that do not express the reporter [99]. Further, time-course analyses did not reveal classical neurogenesis evidenced by place of birth, neuronal differentiation, maturation and integration, raising the possibility that some mature neurons may express Nestin in midbrain [99]. Others have found hints of differentiation by means of cell growth and higher levels of expression of mature neuronal genes over long periods, albeit low proliferative rate and no evidence of migration [100]. Interestingly, single-cell gene expression data suggested that up-regulation of mature neuronal genes in *Nestin*-*cre*^*ERT2*^ -traced midbrain cells occurs over several months, not days, providing robust evidence for presence of Nestin^+^ neural progenitors in adult midbrain. However, controversial findings were described in a model of Parkinson’s disease induced by dopaminergic neuron degeneration driven by 6-hydroxy-dopamine intra-cerebral injection. Here, numbers of *Nestin*-*cre*^*ERT2*^ -traced cells increase by two-fold in substantia nigra, with no changes in the expression on neuronal nuclear antigen. Remarkably, 4 days after tamoxifen many *Nestin*-*cre*^*ERT2*^ -traced cells have mature neuronal morphology, and are positive for both neuronal nuclear antigen and the pluripotency marker Sox-2. This short timeframe may be not enough for Nestin^+^ progenitors to become mature neurons, again raising the possibility that mature neurons express Nestin and other immature genes under certain conditions and in contexts that may be not neurogenic [100]. Other authors have selectively deleted tyrosine hydroxylase in Nestin- or Sox-2-traced cells using *cre*^*ERT2*^ systems. 6 months after the start of a 6 week treatment with tamoxifen, mice with *Nestin*-mediated tyrosine hydroxylase deletion showed lower numbers of tyrosine hydroxylase^+^ neurons in substantia nigra, while no reduction was observed following *Sox-2*-mediated excision. This finding further supports that Nestin-expressing cells regenerate dopaminergic neurons [101]. Previously discussed factors related to limitations of in vivo models may have influenced these controversial results that deserve further investigation and are highly relevant in search for therapeutic targets in Parkinson’s disease.

Cell therapy is an approach extensively pursued experimentally due to its valuable therapeutic potential against neurodegenerative diseases like Parkinson. Endogenous NSCs and progenitors are unable to promote full and long-lasting recovery [102]. Further, the source of cells is a heavy milestone. A potential strategy to overcome this limitation is generation of induced dopaminergic neurons from induced pluripotent stem cells derived from patients with Parkinson. As of today, however, induced dopaminergic neurons differ remarkably from primary midbrain neurons in global gene expression, particularly in genes related to maturation [103]. Others have been able to elicit dopaminergic neuronal conversion from adult fibroblasts derived from healthy donors and Parkinson patients [104]. Numbers of resulting cells seem to be the weakness in this design. An exciting approach is the reprogramming from somatic cells into multipotent NSCs, which offers reduced risk of tumor formation versus induced pluripotent stem cells, and production of higher amounts of homogeneous cell populations as compared to induced neuronal cells. Recently, induced NSCs were reprogrammed from mouse and human fibroblasts with introduction of the gene Sox-2 only [105]. Indeed, resulting cells express Nestin, and resemble NSCs in their morphology, self-renewal, differentiation potential into mature neurons, astrocytes and oligodendrocytes in vitro and in vivo, and gene expression profiles [105]. Others have found that Sox-2 alone shows limited conversion ability from adult human somatic cells [106]. In this work, let-7 microRNA was found critical for formation of paired box 6/NESTIN-positive colonies to immunocytochemically characterize the NSCs induced from human adult fibroblasts, and for their proliferation and self-renewal [106]. Certainly, fine-tuned control of Nestin expression is a key and challenging factor that will provide a significant step forward towards generation of safe and functional induced NSCs suitable for patient therapy.

Scientific endeavor in search for alternative sources of NSCs and progenitors has led to consideration of bone marrow mesenchymal cells, with varying degree of success. These cells are currently being tested in exploratory clinical trials against multiple sclerosis (i.e., NCT01854957), major cause of neurological disability with fast progression and young adult onset [107]. Immune cells targeting self-antigens in the central nervous system in genetically susceptible individuals are thought to play a key role in loss of myelin and myelinating cells and subsequent damage to axons and neurons, but its true etiology remains elusive [108]. Upon intravenous injection, bone marrow stromal cells successfully infiltrate brain and spinal cord, and migrate towards lesions in multiple sclerosis. The beneficial effects of these cells are not fully understood but may include immunosuppression, neuroprotection, and/or differentiation, among others [107]. Our recent work has evidenced that bone marrow Nestin^+^ MSCs, with specialized HSC niche function, share a common neural crest ontology with neural progenitor cells [50]. In neonates, this fraction of specialized MSCs also contains Schwann cell precursors that can be differentiated into Schwann cells ex vivo [50, 51]. In adults, Nestin^+^ MSCs and NSCs retain a number of similarities like expression of functional core markers, including Nestin, and ex vivo self-renewal ability as measured by sphere formation. Further, MSCs activate the Schwann cell program in vivo as a consequence of the neuroglial damage caused in the bone marrow by mutated HSCs [51]. Hence, it is reasonable to hypothesize that bone marrow Nestin^+^ MSCs may provide a useful source for cell therapy in neurodegenerative diseases. Future work is required to validate this hypothesis.

### Bone marrow

Given the role of Nestin^+^ cells within the healthy HSC niche, contributing to HSC maintenance in the bone marrow and mediating HSC circadian mobilization into the periphery [9, 21, 87, 109], Nestin^+^ cells have recently gained interest in the context of bone marrow pathologies, and particularly hematological malignancies.

In preclinical models of myeloproliferative neoplasms, a subclass of hematological cancer, our recent work has provided evidence on the pathogenic role of bone marrow Nestin^+^ cell alterations, and the promising therapeutic value of their targeting [51]. The acquired somatic mutation in the Janus kinase 2 (JAK2) gene resulting in a valine to phenylalanine substitution at position 617 (JAK2-V617F) is present in the majority of these patients, and renders hematopoietic precursors with proliferative and survival advantages [110]. Using a transgenic mouse model that expresses the human mutant *JAK2*-*V617F* under the endogenous promoter of *Jak2* in an inducible manner, we showed that interleukin-1β produced at early disease stage, at least partially by mutant HSCs, induces damage of neuroglial fibers. Reduced sympathetic regulation together with interleukin-1β stimulation results in *Nestin*-*gfp*^+^ cell apoptosis that then allows expansion of mutant HSCs. Nestin^+^ cell number and *Nestin* messenger RNA expression are also reduced in the bone marrow of patients with myeloproliferative neoplasms. The pathogenic role of *Nestin*-*gfp*^+^ cell reduction was uncovered by enforced reduction of these cells using *Nestin*-*cre*^*ERT2*^ *iDTA* mice, which aggravates hallmarks of disease, including hematological parameters and osteosclerosis of the bone marrow. As previously discussed, selective depletion of Nestin^+^ in vivo using *Nestin*-*cre*^*ERT2*^ *iDTA* mice did not affect Schwann cells but reduced numbers of CD90^+^ mesenchymal cells. Besides, selective deletion of Cxcl12 in *Nestin*-*cre*^*ERT2*^ mice leads to expansion of the HSC compartment, suggesting the contribution of Cxcl12 in control of mutant hematopoietic progenitor numbers. However, potential targeting of Nestin^+^ CD31^+^ endothelial cells cannot be ruled out with these *Nestin*-*cre*^*ERT2*^ strategies and may have contributed to the observed phenotypes. Conversely, compensation of sympathetic regulation on Nestin^+^ cells using beta-3 adrenergic agonist treatment rescues numbers of *Nestin*-*gfp*^+^ and Schwann cells, blocks Schwann cell gene program activation in *Nestin*-*gfp*^+^ cells, improves levels of Cxcl12, ameliorates hallmarks of disease and interleukin-1β levels, and efficiently decreases mutant hematopoietic progenitors with no effect on normal progenitors. Although beta-3 adrenergic receptors are somewhat restricted to selective cell subsets and they are not present in hematopoietic cells, certain types of adipocytes and endothelial cells may show expression in addition to Nestin^+^ cells [111]. Nonetheless, our data suggest that targeting the beta-3 adrenergic receptor with agonist drugs may have clinical implications to improve treatment of patients with myeloproliferative neoplasms, and Nestin^+^ cells contribute to these events [51]. Further, the most important hematopoietic disease-related pain affects to bone, and it was traditionally related to osteolytic lesions and infiltration of bone marrow with malignant cells. Our work may provide hints linking pathogenesis and pain in hematopoietic malignancies through neuroglial damage, in both disease mice and humans, which may contribute to bone pain reported in patients [112]. Sympathetic nervous system compensation with beta-3 adrenergic agonists did not rescue neural fibers in mouse models [51], so it will be interesting to see their ability to relieve pain in patients.

In an MLL-AF9^+^ acute myeloid leukemia model, generated after transduction of Linage^−^c-Kit^+^Sca-1^+^ cells with the MLL-AF9 oncogene and serial transplantation of these cells, neuropathy promotes leukemic bone marrow infiltration [113]. In their model, development of acute myeloid leukemia disrupts neural fibers and the quiescence of Nestin^+^ cells, leading to their expansion by 3.8-fold with osteoblastic priming ex vivo. Transplantation of leukemic cells using recipients *Osterix*-*cre*^*ERT2*^ together with the reporter *tdTomato* to label osteolineage cells showed, under continuous tamoxifen administration via diet, increased bone remodeling with accumulation of osteoblasts and reduction of mature bone-forming osteoblasts [113]. Osterix is expressed earlier than osteocalcin in the osteoblast lineage [114], but still osterix-expressing cells do not represent a pool of progenitors with self-renewal capacity in healthy adult mice [115]. Osterix-labeled cells from leukemic marrow expressed Lepr, unlike mature osteoblast from healthy mice, but participation of Nestin^+^ cells in these processes was only inferred and not confirmed in vivo. NG2^+^ periarteriolar cells are reduced in  acute myeloid leukemia, and this is not likely due to increased differentiation, as no double positive cells for Pdgfrα and CD51 were fate mapped by *NG2*-*cre*^*ERTM*^ with a reporter [113]. Nevertheless, contribution of NG2 traced cells to more differentiated osteoblasts was not studied. Further, reduction of NG2^+^ cells and their expression of HSC-supportive genes was only correlated and not causally related to lower numbers of long-term HSCs. Similarly, the reduced expression of HSC maintenance and retention genes in Nestin^+^ cells was only correlated and not causally related to mobilization of progenitors to circulation and spleen. Nevertheless, the beta-2 adrenergic receptor expressed on stromal cells seems to play a role in acute myeloid leukemia development as higher leukemic bone marrow infiltration occurs in mice lacking this receptor. Conversely, administration of the beta-2 adrenergic agonist Clenbuterol hydrochloride led to reductions of phenotypic leukemic stem cells in bone marrow, spleen, and blood and tended to extend survival. However, Clenbuterol also had a cell-autonomous action in vitro, enhancing proliferation of MLL-AF9 cells [113], which discourages its use as a potential therapeutic target. Future work should address the causal contribution of Nestin^+^ cells in acute myeloid leukemia development, and its potential use as target for novel drug development.

## Conclusions

A large body of evidence points to Nestin as a unique intermediate filament that accompanies self-renewal capacity in several subsets of stem cells and progenitors, particularly those of the neural and mesenchymal lineages. Nestin seems to have an impact on stem cell migration and differentiation, yet the knowledge on the underlying mechanisms is limited. Surprisingly, little is also known on the regulatory signaling pathways that control Nestin expression and function, which should be the focus of future work. The precise identity of Nestin^+^ cells is currently the target of extensive endeavor in in vivo models. Data obtained from these tools are highly valuable, but it should be considered in the context of their weaknesses as well to prevent misinterpretation. In parallel, the mechanistic role and promising therapeutic value of Nestin^+^ cells is also under research in preclinical models of disease, especially neurodegenerative diseases and bone marrow malignancies. Future improved in vivo models and detection tools will help characterize the real nature of Nestin^+^ cells and confirm their application as therapy in those diseases.
